# Accuracy of tongue strength, endurance, and pressure using Iowa oral performance instrument and predictors of dysphagia in community-dwelling older adults: a cross-sectional study

**DOI:** 10.1186/s12877-025-05859-z

**Published:** 2025-03-24

**Authors:** Yen-Fang Chou, Chien-Mei Sung, Yu-Hao Chu, Kai-Jo Chiang, Ruey Chen, Kondwani Joseph Banda, Chiu-Kuei Lee, Melati Fajarini, Kuei-Ru Chou

**Affiliations:** 1 School of Nursing, College of Nursing, Taipei Medical University, No.250, Wu-Hsing Street, Taipei, Taiwan; 2 Department of Nursing, New Taipei Municipal TuCheng Hospital (Built and Operated by Chang Gung Medical Foundation), New Taipei City, Taiwan; 3 Department of Gerontology and Health Care Management, Chang Gung University of Science and Technology, Taoyuan, Taiwan; 4 Department of Nursing, Fu Jen Catholic University Hospital, Fu Jen Catholic University, New Taipei City, Taiwan; 5 School of Nutrition and Health Sciences, College of Nutrition, Taipei Medical University, Taipei, Taiwan; 6 Department of Nursing, Tri-Service General Hospital, Taipei, Taiwan; 7 School of Nursing, National Defense Medical Center, Taipei, Taiwan; 8 Department of Nursing, Taipei Medical University-Shuang Ho Hospital, New Taipei City, Taiwan; 9 Post-Baccalaureate Program in Nursing, College of Nursing, Taipei Medical University, Taipei, Taiwan; 10 Endoscopy Unit, Department of Surgery, Kamuzu Central Hospital, Lilongwe, Malawi; 11 Faculty of Nursing, Universitas Muhammadiyah Jakarta, DKI Jakarta,, Indonesia; 12 Research Center in Nursing Clinical Practice, Wan Fang Hospital Taipei Medical University, Taipei, Taiwan; 13 Psychiatric Research Center, Taipei Medical University Hospital, Taipei, Taiwan; 14 Research Center for Neuroscience, Taipei Medical University, Taipei, Taiwan

**Keywords:** Accuracy, Oropharyngeal dysphagia, Iowa oral performance instrument, Community-dwelling older adults

## Abstract

**Background:**

Decreased tongue strength, pressure, and endurance are key indicators in determining oropharyngeal dysphagia (OD). This study aimed to examine the accuracy of the Iowa Oral Performance Instrument (IOPI) in assessing tongue strength, endurance, and pressure, and to identify predictors of OD.

**Methods:**

In this study, we analyzed data of community-dwelling older adults (age ≥ 65 years) collected between March to December 2022. The accuracy for IOPI was examined with Receiver operating characteristic curve using area under the ROC curve (AUC), sensitivity (Se) and specificity (Sp) and optimal cutoff with Youden index (J). Bivariate and multivariate logistic regression analysis for predictors of OD were performed presenting odds ratio (OR) with 95% confidence interval (CI).

**Results:**

The cohort consisted of 85 older adults with mean age of 83.25 years (SD 6.76), of which 64 (75.3%) were female. The prevalence of OD using EAT-10 was 8.3%. Tongue strength demonstrated better diagnostic accuracy using anterior tongue strength (ATS): cut-off: 37.5 kPa (AUC: 0.79, Se: 0.86, and Sp: 0.65) and posterior tongue strength: cut-off: 31.5 kPa (AUC: 0.73, Se: 0.71, and Sp: 0.79). Tongue endurance demonstrated better diagnostic accuracy using anterior endurance target second (ATE-Target Sec): cut-off: 2.4 (AUC: 0.96, Se: 0.86, and Sp: 0.90), PTE-Target Sec: cut-off: 1.7 (AUC: 0.93, Se: 0.86, and Sp: 0.83), ATE-Target Max with cut-off: 34.4 kPa (AUC 0.81, Se = 0.86, and Sp = 0.64), and PTE-Target Max with cut-off: 29.5 kPa (AUC: 0.77, Se = 0.86, and Sp = 0.69). Tongue pressure revealed limited diagnostic accuracy using saliva swallowing pressure with cut-off: 23.3 kPa (AUC: 0.60) and effortful swallowing pressure with cut-off: 28.5 kPa (AUC: 0.62). Significant predictors for OD were frailty (3.02, 95%CI: 1.56–5.88), age (1.17, 95%CI: 1.01–1.35), nutritional status (0.72, 95%CI: 0.57–0.92), ATS (0.86, 95%CI: 0.77–0.97), ATE-Target Max (0.90, 95%CI: 0.84–0.97), PTE-Target Max (0.92, 95%CI: 0.86–0.99), ADL (0.91), IADL (0.67), and depression (1.32).

**Conclusions:**

The findings suggest that tongue strength and endurance, measured by IOPI, are more effective parameters than tongue pressure, with frailty, age, nutritional status, ATS, ATE-Target Max, PTE-Target Max, ADL, IADL, and depression being essential for early screening of OD in community-dwelling older adults.

**Clinical trial number:**

Not applicable.

**Supplementary Information:**

The online version contains supplementary material available at 10.1186/s12877-025-05859-z.

## Introduction

Oropharyngeal dysphagia (OD) is characterized by impairment in the oral cavity, pharynx, and upper esophageal phases, recognized as a geriatric syndrome by the European Society for Swallowing Disorders and European Union Geriatric Medicine Society [1, 2]. Population aging, combined with increased incidence of diseases among older adults, has contributed to the increase in the prevalence of OD in this population. According to Rajati et al., [3] the global prevalence of OD is estimated at 43.8% in the general population with significant variations across care settings: 50.2% in nursing homes, 42.5% in rehabilitation facilities, 36.5% in hospitals [4], and 29.0% in geriatric hospitals [5]. These variations are attributable to the demographics of older adults, OD evaluation time, setting, and assessment tools [3–5]. Consequently, screening for OD in community-dwelling older adults is crucial for early identification of dysphagia-associated complications.

Previous research has identified several physical, physiological, and psychosocial factors that are associated with functional decline in older adults, which significantly contribute to the onset of dysphagia [6–11]. Frailty in older adults is associated with dysphagia due to reduced physiological reserve and muscle deterioration [6]. Additionally, age-related decline in muscle mass and function of the swallowing muscles increases the risk of dysphagia [7]. Nutritional status of older adults, particularly malnutrition, causes weakened swallowing muscles, further increasing the risk of dysphagia [2–5, 8]. Decreased tongue strength and endurance, which are key indicators of impaired swallowing, have been shown to increase the risk of aspiration [9]. Furthermore, impaired physical function, indicative of reduced activities of daily living (ADL) and instrument ADL (IADL) performance, contributes to development of dysphagia demonstrating overall functional decline, including swallowing muscles [10]. As such, routine assessment of demographic characteristics can enhance early detection and management of OD in older adults.

Despite the serious complications associated with OD, such as aspiration pneumonia and malnutrition, it remains under-reported and often undetected or untreated in community settings due to the lack of reliable and accessible screening methods [12]. While subjective screening tools, such as the Eating Assessment Tool-10 (EAT-10), offer a valuable means for early detection of OD, the objective assessment of tongue function using the Iowa Oral Performance Instrument (IOPI) has also emerged as a crucial tool in screening for OD [12–14]. The IOPI, a portable handheld device that measures the pressure exerted on an air-filled bulb, provides quantifiable data on tongue strength, endurance, and pressure, which are key factors in bolus propulsion during swallowing [14–15]. However, despite the ease of use of subjective screening tools and the objectivity offered by the IOPI, the utilization of swallowing screening tools in the community remains limited [16]. Additionally, the use of gold-standard assessments, such as videofluoroscopic swallowing study (VFSS) and fiber-optic endoscopic evaluations of swallowing (FEES), is often impractical in community settings due to their need for specialized equipment and trained personnel. This limitation often leads to underdiagnosis or missed diagnosis of oropharyngeal dysphagia (OD) among community-dwelling older adults. Given the potential of objective tongue measurements to enhance early detection of OD, integrating these tools into routine screening protocols could significantly improve the identification and management of OD in older adults. Therefore, the primary objective of this study was to (1) evaluate the accuracy and determine optimal cut-off points for IOPI measurements of tongue strength, endurance, and pressure in detecting OD, and (2) identify predicting factors for OD among community-dwelling older adults.

## Methods

### Study design and participant selection

In this cross-sectional study, patient enrollment was conducted from March to December 2022. The study was performed according to the Strengthening the Reporting of Observational Studies in Epidemiology (STROBE) checklist: cross-sectional studies guidelines [17].

We employed a purposive sampling technique to enroll community-dwelling older adults in Taiwan. The enrolled participants were aged ≥ 65 years; were able to listen, speak, read, and write Mandarin; volunteered to participate in the study; and signed the informed consent form (ICF). The exclusion criteria for this study were as follows: (1) significant frailty and required support to stand or walk (ADL score, sub-items: Transfer and Mobility on level surfaces 5 points or less) (2) severe communication difficulties; (3) use of anticholinergics, benzodiazepine, or antihistamines; (4) tracheotomy or history of oral or pharyngeal surgery; (5) Neuromuscular disorders (e.g., Parkinson’s disease) or Dementia, cerebrovascular disease (e.g., stroke), decreased sensory and motor function of oral and facial muscles, or dysphagia related diseases. The CONSORT 2010 flow diagram in Figure S9 depicts the participant selection.

### Data collection

In the initial stage, researchers underwent training for conducting swallowing evaluations. Participant enrollment was performed after the consistency of the swallowing evaluation among researchers was confirmed. After explaining the purpose of the study, screening inclusion and exclusion criteria, participants completed the Informed Consent Form (ICF), with a copy retained by both the researcher and the participant. Another researcher then assisted the participants in completing the questionnaire, and the IOPI was measured by a trained assessor.

### Demographic variables and instruments

Demographic information, including chronic disease status, was obtained using the following tools: EAT-10 [18], Iowa Oral Performance Instrument (IOPI) [19], Oral Health Assessment Tool (OHAT) [20], Barthel index and instrumental activities of daily living (IADL) for physical function evaluation [21, 22], Cardiovascular Health Study (CHS) for frailty evaluation [23], Mini-Nutritional Assessment (MNA) for nutritional assessment [24], Mini-Mental Status Examination (MMSE) for cognitive function [25], The PAR authorization was obtained to use MMSE in the current study, and Geriatric Depression Scale (GDS) for emotional problem [26].

### Demographic information

This study collected information on each participant’s age, sex, marital status, education level, residential status, and chronic disease status.

### Swallowing function evaluation tools

(a) Eating Assessment Tool-10.

The EAT-10 is a self-rated questionnaire used to report the symptoms of OD [18]. It comprises 10 items used to evaluate various OD symptoms, clinical characteristics, psychological status, and social influence. Each item is scored on a scale of 0 to 4, with 0 indicating no problem and 4 indicating severe problems. The EAT-10 has a total possible score of 40, with a total score of ≥ 3 indicating swallowing abnormalities. A higher EAT-10 score indicates more severe OD. The EAT-10 has a sensitivity of 89% and a specificity of 82% for OD detection [27, 28], and it has been translated into many languages. The internal consistency (Cronbach’s α) of the different versions of the EAT-10 is 0.84–0.96; the ICC is 0.70–1.00 [13], the test–retest reliability is > 0.7, and the interrater reliability is > 0.7 [29]. In this study, as per the recommendations of Zhang et al., [13] a cutoff point of ≥ 3 was used to indicate swallowing abnormalities with sensitivity, specificity, and area under the curve (AUC) of using VFSS and FEES being 0.87, 0.82, and 0.90, respectively.

(b) Iowa Oral Performance Instrument (IOPI).

The Iowa Oral Performance Instrument (IOPI) (IOPI Medical, Redmond, WA, USA) is a portable handheld device that measures tongue function by tongue (i) strength, (ii) endurance, and (iii) pressure following the manufacturer’s guidelines [14, 15, 19, 30, 31]. IOPI consists of a pressure ball, a connecting tube and a main body. The bulb is made of soft rubber, approximately 3.5 cm long and 1.5 cm wide, filled with approximately 2.8 ml of air, and connected to the pressure port of the main body. Each muscle force was measured three times at 1-min intervals, and the maximum value (kPa) was recorded [31]. The position of the stress ball will vary depending on the muscle being measured. Before administering the test, the assessor will explain each step one by one and let the subjects practice to confirm the correct position.

(i) Tongue strength is the maximum pressure generated by the tongue pressing a standard-sized air-filled bulb against the palate measured by anterior tongue strength (ATS) and posterior tongue strength (PTS). (1) To measure ATS, position the ball longitudinally on the participant’s hard palate, just posterior to the alveolar ridge. Then, ask the participant to gently close their lips and press the bulb as hard as possible with the front of their tongue for 2 s [31]. (2) To measure PTS, place the tip of the bulb at the transition between the hard palate and the soft palate. The tube should be placed gently between the front teeth. During the task, the mandible should remain intrinsically stable (i.e., the jaw should not open and close but remain quietly stable) and the bulb should be pressed as hard as possible with the back of the tongue for 2 s [31]. ATS and PTS measurements are expressed in kilopascals (kPa) with average maximum pressure of 60 kPa ranging between 40 and 80 kPa in older adults. IOPI shows strong reliability and validity for tongue strength, with ICC values ranging from 0.91 to 0.98 and correlation coefficients (r) ranging from 0.70 to 0.80, respectively [14, 15, 19].
(ii) Tongue endurance is the length of time in seconds to maintain 50% of maximum pressure with target value set to 50% of the individual’s maximum pressure and the timing of how long the individual can hold on is measured. The target value is calculated as: *T = P*_*max*_ x (*E*/100) where *T* = Target value, *P*_*max*_ = maximum tongue pressure, *E* = Effort (%). Anterior tongue endurance target maximum (ATE-Target Max) and posterior tongue endurance target maximum (PTE-Target Max) values are measured. The time of anterior and posterior tongue endurance target second (ATE-Target and PTE-Target Sec) below 10 s is considered impaired indicating weakened oral musculature, increasing the risk of dysphagia. IOPI shows strong reliability and moderate validity for tongue endurance, with ICC values ranging from 0.85 to 0.90 and correlation coefficients (r) ranging from 0.60 to 0.70, respectively [14, 15, 19].
(iii) Tongue pressure is the maximum pressure generated by the tongue pressing an IOPI standard-sized air-filled bulb against the palate when swallowing. Saliva swallowing pressure (SSP): The pressure ball is used to measure the position of the anterior tongue strength. Then, the participant is asked to gently close his lips and swallow his saliva, is the pressure generated during a normal saliva swallow. Effortful swallowing pressure (ESP): The pressure ball is used to measure the anterior tongue strength position. Then, the participant is asked to put his tongue between the front teeth or outside the mouth and swallow forcefully, is the pressure generated during an effortful swallow [31]. SSP values below 20 kPa and ESP values below 30 kPa in older adults are considered sub-optimal for safe swallowing. IOPI shows strong reliability and validity for tongue pressure, with ICC values ranging from 0.90 to 0.94 and correlation coefficients (r) ranging from 0.70 to 0.85 [19, 30, 32]. The IOPI is a valid and reliable assessment tool for tongue function.

### Data analysis

Data analysis was conducted in SPSS 25.0. For descriptive statistics, independent *t* test was used to analyze continuous variables with normal distribution according to the Shapiro–Wilk normality test. The Mann–Whitney *U* test was employed for continuous variables with non-normal distribution. The chi-squared test was used to examine categorical variables. Variables with a P-value of < 0.20 in the binary logistic regression analysis were included in the multivariate logistic regression using the Forward-Step (Conditional) method, ensuring that only the most statistically significant predictors were retained in the final model [33]; odds ratios (ORs) were calculated using multivariate logistic regression to identify the factors with significant correlations with OD [33]. The receiver operating characteristic (ROC) curve and Youden index (J) were also employed [34, 35]. The range of the AUC scores is 0.5–1.0, with an AUC of 0.5 considered to indicate pure chance and an AUC of 1.0 considered to denote optimal prediction accuracy. AUCs of 0.50–0.59, 0.60–0.69, 0.70–0.79, 0.80–0.89, and 0.90–1.0 were considered to indicate low or extremely poor accuracy, poor accuracy, fair accuracy, good accuracy, and extremely good accuracy, respectively [34]. All AUC values were expressed as 95% CIs. The Youden index with acceptable values of 0.5–1.0 was used to identify the optimal cutoff point. A difference with *P* < 0.05 was considered significant.

### Study privacy and data confidentiality

This study was approved by the Institutional Review Board of Chang Gung Hospital (Approval No.: 202002633B0C501). Due to ethical considerations, all collected data was anonymously coded and entered into a computer for encryption to ensure the rights and privacy of the study participants would be protected.

### Role of the funding source

There was no role of the study funders in the study design, data collection, data analysis, data interpretation, and writing of the manuscript.

## Results

### Demographic characteristics and prevalence of OD in older adults

A total of 85 community-dwelling older adults were recruited into this study. The Prevalence of OD was 8.3% for the EAT-10 (score ≥ 3, *n* = 7). The tongue pressure measurement of the IOPI revealed that the mean anterior tongue strength (ATS), posterior tongue strength (PTS), anterior and posterior tongue endurance target second (ATE-Target and PTE-Target Sec), and anterior and posterior tongue endurance target maximum (ATE-Target Max and PTE-Target Max) in the OD group were lower than those in the non-OD group. Significant difference was observed between the groups for tongue strength (ATS, *P* = 0.012; PTS, *P* = 0.042) and tongue endurance (ATE-Target Sec, *P* < 0.001; PTE-Target Sec, *P* < 0.001; ATE-Target Max, *P* = 0.006; PTE-Target Max, *P* = 0.02) (Table 1).

**Table 1:** Baseline characteristics of participants in OD (*N* = 85)

Characteristics	Total (*N* = 85)	Swallowing Function		Z	*P* value
		Normal	Abnormal		
EAT-10, No. (%)	85 (100.0)	78 (91.7)	7 (8.3)		
IOPI-TP Mean (SD)‡					
ATS	41.4 (10.63)	42.3 (10.28)	31.4 (9.84)	−2.51	**0.012**
PTS	40.8 (10.63)	41.5 (10.67)	33.3 (6.95)	−2.03	**0.042**
ATE-Target Sec	7.7 ( 6.92)	8.4 (6.88)	0.7 (0.84)	−4.05	**0.001**
PTE-Target Sec	4.0 ( 3.20)	4.3 (3.15)	0.6 (0.63)	−3.77	**0.001**
ATE-Target Max	36.9 (12.70)	38.1 (12.13)	23.5 (11.87)	−2.73	**0.006**
PTE-Target Max	34.0 (12.91)	35.1(12.57)	22.8 (12.11)	−2.32	**0.020**
SSP	30.7 (11.52)	31.0 (11.34)	26.9 (13.69)	−0.90	0.366
ESP	33.2 (12.41)	33.7 (12.24)	28.2 (14.18)	−1.04	0.299

Note

Mean (SD);   ‡ Mann–Whitney U test; if *P < 0.05 indicated in **bold**

Abbreviations: EAT-10, Eating Assessment Tool-10; a total score of ≥ 3 indicates abnormality; OD, Oropharyngeal Dysphagia; ATS, anterior tongue strength; PTS, posterior tongue strength; ATE-Target Sec, anterior tongue endurance target second; PTE-Target Sec, posterior tongue endurance target second; ATE-Target Max, anterior tongue endurance target maximum; PTE-Target Max, posterior tongue endurance target maximum; SSP, saliva swallowing pressure; ESP, effortful swallowing pressure

Regarding demographic characteristics, the mean age was 83.25 years (standard deviation [SD] = 6.76), 75.3% of the participants were women, and 72.9% lived alone. All participants had a chronic disease, and the mean number of chronic diseases was 3.28. The chronic diseases included hypertension, hyperlipidemia, sleep disorder, cataract, heart disease, diabetes mellitus, osteoporosis, benign prostate hyperplasia, mild cognitive impairment, arthritis, depression, kidney disease, and glaucoma. No significant differences were noted in age, sex, education level, marital status, residential status, and chronic disease status between the OD and non-OD groups (Table 2; Table S1).

**Table 2:** Baseline characteristics of participants with OD in cohort (*N* = 85)

Characteristics	Total (*N* = 85)	Swallowing Function		t/χ^2^	*P* value
		Normal	Abnormal		
Age, mean (SD)^a^	83.25 (6.76)	82.74 (6.75)	88.86 (4.10)	t = 2.350	0.252
Sex, No. (%)^b^				χ^2^ = 3.58 (1)	0.058
Male	21 (24.7)	17 (20.2)	4 (57.1)		
Female	64 (75.3)	61 (71.8)	3 (42.9)		
BMI (kg/m^2^), mean (SD)^a^	23.89 (3.05)	23.99 (2.73)	22.06 (5.57)	t = − 0.905	0.399
Marital status, No. (%)^c^					0.821
Unmarried	3 (3.5)	3 (3.8)	0 (0.0)		
Married	32 (37.6)	30 (38.5)	2 (28.6)		
Widowed or Divorced	50 (58.9)	45 (57.7)	5 (71.4)		
Education level, No. (%)^c^					0.769
Elementary school and below	16 (18.8)	14 (17.9)	2 (28.6)		
Junior high to high school	38 (44.7)	36 (46.2)	2 (28.6)		
College/university	31 (36.5)	28 (35.9)	3 (42.9)		
Residential status, No. (%)^c^					0.382
Alone	62 (72.9)	58 (74.4)	4 (57.1)		
Living with family members	23 (27.1)	20 (25.6)	3 (42.9)		
No. of chronic illnesses, mean (SD)^a^	3.28 (1.73)	3.23 (1.69)	3.86 (2.27)		0.201
ADL, mean (SD)‡	95.59 (8.91)	96.73 (6.88)	82.86 (17.28)	−3.399	**0.001**
IADL, mean (SD)‡	6.82 (1.65)	6.95 (1.60)	5.43 (1.72)	−2.524	**0.012**
FS, mean (SD)‡	1.29 (1.24)	1.13 (1.10)	3.14 (1.35)	−3.420	**0.001**
MNA, mean (SD)‡	26.38 (2.63)	26.65 (2.21)	23.36 (4.81)	−1.677	0.094
GDS, mean (SD)‡	2.72 (3.19)	2.38 (2.81)	6.43 (4.86)	−2.099	**0.036**
MMSE, mean (SD)‡	26.91 (4.09)	27.13 (3.91)	24.43 (5.53)	−1.745	0.081
OHAT, mean (SD)‡	1.58 (1.64)	1.55 (1.63)	1.86 (1.77)	−0.528	0.598

Note: Mean (SD); ^a^ Independent *t* test; ^b^ Person χ^2^ (chi-squared test); ^c^ Fisher’s exact test (chi-squared test); ‡ Mann–Whitney *U* test; if *P* < 0.05 indicated in **bold**

Abbreviations: EAT-10, Eating Assessment Tool-10; a total score of ≥ 3 indicates abnormality; OD, Oropharyngeal Dysphagia; ADL, activities of daily living; IADL, instrumental activities of daily living; FS, frailty status; MNA, Mini-Nutrition Assessment; GDS, Geriatric Depression Scale; MMSE, Mini-Mental State Examination; OHAT, Oral Health Assessment Tool

### Demographic characteristics between OD and non-OD groups

The Mann–Whitney *U* test was used to compare the various health indices between the OD and non-OD groups. The results (Table 2) revealed significant differences in the Barthel index score (*P* = 0.001), IADL score (*P* = 0.012), frailty (*P* = 0.001), GDS score (*P* = 0.036). The OD group had significantly poorer scores than the non-OD group did in terms of the aforementioned health indices, and OD severity affected ADL in the older adults.

The mean overall MNA score was lower in the OD group than in the non-OD group, but the difference was non-significant (*P* = 0.094). The mean MMSE score was lower in the OD group than in the non-OD group, but the difference was non-significant (*P* = 0.081). Regarding oral health, the difference in the OHAT score between the two groups was non-significant (*P* = 0.598) (Table 2).

### Accuracy and optimal cut-off points for tongue strength, endurance, and pressure using the IOPI

Regarding tongue strength, the results for anterior tongue strength (ATS) were: AUC = 0.79, 95%CI = 0.59–0.99 with optimal cut-off point at 37.5 kPa, J = 0.51, sensitivity = 0.86, and specificity = 0.65 (*P* = 0.012) (Table 3; Figure S1; Table S2). The results for posterior tongue strength (PTS) were: AUC = 0.73, 95%CI = 0.58–0.89 with optimal cut-off point at 31.5 kPa, J = 0.51, sensitivity = 0.71, and specificity = 0.79 (*P* = 0.042) (Table 3; Figure S2; Table S3). The results indicate that ATS and PTS have better diagnostic accuracy for OD screening. Thus, the results suggest that tongue strength has better diagnostic ability to detect OD in community-dwelling older adults.

**Table 3:** Performance of the IOPI parameters for OD screening (*N* = 85)

Scale/TP	AUC	Asymptotic 95% CI		Cut-off	Youden Value	Sensitivity	Specificity	*P* value
		Lower Bound	Upper Bound					
ATS	0.79	0.59	0.99	37.5	0.51	0.86	0.65	**0.012**
PTS	0.73	0.58	0.89	31.5	0.51	0.71	0.79	**0.042**
ATE-Target Sec	0.96	0.92	1.00	2.4	0.76	0.86	0.90	**0.001**
PTE-Target Sec	0.93	0.87	0.99	1.7	0.69	0.86	0.83	**0.001**
ATE-Target Max	0.81	0.68	0.95	34.4	0.50	0.86	0.64	**0.006**
PTE-Target Max	0.77	0.61	0.92	29.5	0.55	0.86	0.69	**0.02**
SSP	0.60	0.35	0.85	23.3	0.29	0.57	0.72	0.366
ESP	0.62	0.37	0.87	28.5	0.19	0.57	0.62	0.299

a. Under the nonparametric assumption; if *P* < 0.05 indicated in **bold**

b. Null hypothesis: true area = 0.5

c. Abbreviations: IOPI: Iowa Oral Performance Instrument; OD, Oropharyngeal Dysphagia; AUC, Area under the ROC curve; ATS, anterior tongue strength; PTS, posterior tongue strength; ATE-Target Sec, anterior tongue endurance target second; PTE-Target Sec, posterior tongue endurance target second; ATE-Target Max, anterior tongue endurance target maximum; PTE-Target Max, posterior tongue endurance target maximum; SSP, saliva swallowing pressure; ESP, effortful swallowing pressure

Regarding tongue endurance, the results for ATE-Target Sec were: AUC = 0.96, 95%CI = 0.92–1.00 (*P* = 0.001) with optimal cut-off point at 2.4, J = 0.76, sensitivity = 0.86, and specificity = 0.90 (Table 3; Figure S3; Table S4). The results for PTE-Target Sec were: AUC = 0.93, 95%CI = 0.87–0.99 (*P* = 0.001) with optimal cut-off point at 1.7, J = 0.69, sensitivity = 0.86, and specificity = 0.83 (Table 3; Figure S4; Table S5). The results indicate that ATE-Target Sec and PTE-Target Sec have better diagnostic accuracy for OD screening in community-dwelling older adults. Similarly, the results for ATE-Target Max were: AUC = 0.81, 95%CI = 0.68–0.95 (*P* = 0.006) with optimal cut-off point at 34.4 kPa, J = 0.50, sensitivity = 0.86, and specificity = 0.64 (Table 3; Figure S5; Table S6). The results for PTE-Target Max were: AUC = 0.77, 95%CI = 0.61–0.92 (*P* = 0.02) with optimal cut-off point at 29.5 kPa, J = 0.55, sensitivity = 0.86, and specificity = 0.69 (Table 3; Figure S6; Table S7). The results indicate that ATE-Target Max and PTE-Target Max have better diagnostic accuracy for OD screening. Thus, the results suggest that tongue endurance using ATE-Target and PTE-Target Sec and Max has better diagnostic ability to detect OD in community-dwelling older adults.

Regarding tongue pressure, the results for saliva swallowing pressure (SSP) were: AUC = 0.60, 95%CI = 0.35–0.85 with optimal cut-off point at 23.3 kPa, sensitivity = 0.57, and specificity = 0.72 (Table 3; Figure S7; Table S8). The results for effortful swallowing pressure (ESP) were: AUC = 0.62, 95%CI = 0.37–0.87 with optimal cut-off point at 28.5 kPa, sensitivity = 0.57, and specificity = 0.62 (Table 3; Figure S8; Table S9). The results indicate that tongue pressure using SSP and ESP has limited diagnostic accuracy for OD screening in community-dwelling older adults.

### Predicting factors of OD

In this study, the univariate logistic regression revealed that the predicting factors of OD in community-dwelling older adults were frailty (OR, 3.02, 95%CI: 1.56–5.88), age (OR, 1.17, 95%CI: 1.01–1.35), nutritional status (OR, 0.72, 95%CI: 0.57–0.92), ATS (OR, 0.86, 95%CI: 0.77–0.97), ATE-Target Max (OR, 0.90, 95%CI: 0.84–0.97), PTE-Target Max (OR, 0.92, 95%CI: 0.86–0.99), ADL (OR, 0.91), IADL (OR, 0.67), and depression (OR, 1.32). The multivariate logistic regression analysis, after adjusting for age, ADL, IADL, MNA, depression, ATS, PTE-Target Max, revealed that frailty (OR, 2.87, 95%CI: 1.41–5.86) and ATE-Target Max (OR, 0.92, 95%CI: 0.84–0.99) were strong predicting factors of OD (Table 4). The findings demonstrated that frailty, age, nutritional status, ATS, ATE-Target Max, PTE-Target Max, ADL, IADL, and depression were significant predictor factors for OD. In addition, frailty and ATE-Target were strong predictors for OD.

**Table 4:** Bivariate and multivariate logistic regression model of OD (*N* = 85)

Characteristics	β (SE)	Wald	*P* value	OR	95% CI
**Bivariate LR regression**					
AGE	0.16 (0.07)	4.67	**0.031**	1.17	1.01–1.35
Total number of chronic diseases	0.20 (0.22)	0.83	0.363	1.22	0.79–1.88
ADL	−0.10 (0.03)	7.59	**0.006**	0.91	0.85–0.97
IADL	−0.39 (0.18)	4.56	**0.033**	0.67	0.47–0.97
FS	1.11 (0.34)	10.64	**0.001**	3.02	1.56–5.88
MNA	−0.33 (0.12)	7.20	**0.007**	0.72	0.57–0.92
MMSE	−0.11(0.07)	2.45	0.118	0.89	0.78–1.03
GDS	0.28 (0.10)	7.64	**0.006**	1.32	1.08–1.61
OHAT	0.10 (0.22)	0.23	0.635	1.11	0.72–1.71
ATS	−0.14 (0.06)	5.86	**0.015**	0.86	0.77–0.97
PTS	−0.08 (0.04)	3.57	0.059	0.92	0.85–1.00
SSP	−0.03 (0.04)	0.82	0.365	0.97	0.90–1.04
ESP	−0.04 (0.04)	1.24	0.266	0.96	0.90–1.03
ATE-Target Max	−0.10 (0.04)	7.00	**0.008**	0.90	0.84–0.97
PTE-Target Max	−0.08 (0.03)	5.20	**0.023**	0.92	0.86–0.99
**Multivariate LR Forward-STEP (COND) method**					
Constant	−1.96 (1.52)	1.65	0.198	0.14	
FS	1.06 (0.36)	8.41	**0.004**	2.87	1.41–5.86
ATE-Target Max	−0.09 (0.04)	4.13	**0.042**	0.92	0.84–0.99

Note. Dependent variable = EAT-10, Eating Assessment Tool-10; Dysphagia Screening Scale. The Omnibus test χ^2^(1) = 19.336, *P* < 0.000; −2 log likelihood = 29.02; Cox and Snell R^2^ = 0.203; Nagelkerke R^2^ = 0.469; Hosmer and Lemeshow test χ^2^(7) = 7.21, *P* = 0.407; Overall percentage correct = 94.1; if *P* < 0.05 indicated in **bold**

Abbreviations: OD, Oropharyngeal Dysphagia; LR, Logistic regression; ADL, activities of daily living; IADL, instrumental activities of daily living; FS, frailty status; MNA, Mini-Nutrition Assessment; MMSE, Mini-Mental State Examination; GDS, Geriatric Depression Scale; OHAT, Oral Health Assessment Tool; ATS, anterior tongue strength; PTS, posterior tongue strength; ATE-Target Max, anterior tongue endurance target maximum; PTE-Target Max, posterior tongue endurance target maximum

## Discussion

### Prevalence of OD in community-dwelling older adults

In this study, the Prevalence of OD was estimated at 8.3% when an EAT-10 score of ≥ 3. This prevalence rate is significantly lower than that (46%) reported by Banda et al. in older adults aged 60 years and older []. In addition, another meta-analysis revealed that the prevalence of OD in older adults in hospitals, rehabilitation facilities, and nursing homes was 36.5%, 42.5%, and 50.2%, respectively [4]. This variation may be attributed to differences in screening methods, sample characteristics, and setting across studies. Although the gold standard diagnostic tools can be used to determine the prevalence of OD in clinical setting [4], screening tools may be preferable for evaluating OD in community-dwelling older adults because of their feasibility and ease of implementation in such contexts. Although the EAT-10 is the most commonly used subjective screening tool for oropharyngeal dysphagia (OD) [13], it is important to acknowledge its limitations. Being a self-administered questionnaire, EAT-10 can be biased due to the subjectivity of responses. Optimistic participants with dysphagia might under-report their symptoms, leading to misclassification into the non-dysphagia group, while pessimistic or overly cautious individuals might over-report symptoms, resulting in false positives. This bias could affect the accuracy of prevalence estimates and potentially impact the conclusions about the effectiveness of interventions. Therefore, while useful, the EAT-10 should not be considered a definitive diagnostic tool for OD. However, as a reference for initial screening for OD in clinical practice, especially in settings with limited resources, the use of self-report tools including EAT-10 offer a viable alternative. Therefore, the integration of objective assessment tools such as IOPI for detection of OD to ensure comprehensive assessment deserves more attention as aging-related diseases may increase the risk of OD and worsen healthcare burden in community-dwelling older adults.

### Iowa oral performance instrument parameters and OD screening

The results of this study, showing high sensitivity and specificity with cutoff points of 37.5 kPa for anterior tongue strength and 31.5 kPa for posterior tongue strength, further enhance the utility of tongue strength as a better diagnostic tool for screening OD in community-dwelling older adults. Compared to the reported normal values for healthy older adults, which range from 40 to 80 kPa with an average of 56 kPa [31], the cut-off points found in this study are notably lower. This discrepancy can likely be attributed to the participants’ average age of 83.25 years in this study. Age-related physiological changes, including decreased muscle strength, diminished neural control, and overall functional decline [3, 5, 14], are known to impact swallowing function. Therefore, the cut-off points derived from this study are particularly relevant for the older population in community settings, whose tongue strength and swallowing abilities may already be compromised due to age-related physiological deterioration. These results specifically highlight the unique needs and challenges faced by the super-aged society.

The results also demonstrated that tongue endurance using ATE-Target and PTE-Target Sec and Max by IOPI as a parameter with strong ability to accurately detect OD. The normal tongue endurance values range between 15 and 35 s in healthy older adults, with significant declines showing compromised muscle function [31, 36]. However, tongue endurance in older adults with OD might be significantly reduced due to neurogenic or structural impairments that weaken swallowing muscles affecting the ability to maintain sustained pressure during repeated swallowing, leading to premature fatigue and increased risk of aspiration. The decrease in tongue endurance further contributes to the inefficiency in safe transit of bolus and clearance during swallowing, predisposing older adults to penetration and aspiration. The identification of optimal cut-off points of 34.4 kPa for ATE-Target Max and 29.5 kPa for PTE-Target Max in this study, provides reliable benchmarks for distinguishing between OD and non-OD in the community. Moreover, the high accuracy of tongue endurance measurements using ATE-Target Max, and PTE-Target Max provide an accurate assessment of swallowing function, capturing the sustained and coordinated muscle activity required for safe swallowing.

The results also revealed that tongue pressure including SSP and ESP has limited diagnostic ability to detect OD. Tongue pressure measurements have limited ability to capture the functional demands of swallowing making less effective in detecting OD [37, 38]. The average anterior tongue pressure ranges between 34 kPa and 80 kPa in healthy older adults aged ≥ 60 years with the posterior tongue pressure being slightly 5–10% lower [31]. In older adults with OD, reduced tongue pressure impairs the ability to generate adequate intraoral pressure for bolus propulsion leading to poor coordination of swallowing, increasing the risk of residue in the oropharynx and risk of penetration and aspiration. Tongue pressure also lack the precision needed to assess the sustained and coordinated muscle activity necessary for safe swallowing limiting their diagnostic utility [39].

On the other hand, tongue strength and endurance ability to generate and maintain consistent pressure over time, make them crucial parameters for effective and safe swallowing compared to tongue strength and pressure [14, 36]. Overall, the IOPI (i) provides precise and reproducible objective measurements of tongue strength, endurance, and pressure, which are key indicators of swallowing function helping to identify declines in muscle performance and (ii) allows for rapid assessment and ongoing monitoring of tongue function over time making it suitable for repeated assessments. Thus, IOPI can be a reliable, easy to use, and feasible objective screening tool for detecting OD in community-dwelling older adults. However, due to medical regulatory restrictions in different countries, the JMS tongue pressure measurement device (TPM-01, JMS Co. Ltd., Hiroshima, Japan) is widely used in Japan to measure tongue pressure. Previous research has shown that the IOPI and JMS have significant correlations in total cohorts, and male and female participants, separately [40]. Moreover, the development of new technologies has made jaw-opening force, a useful index for assessing dysphagia providing numerical measure of swallowing ability and does not require specialized equipment or environments compared to FEES or VFSS [41]. This offers more diverse and convenient options for measuring tongue pressure in community-dwelling elderly.

### Predictor factors of OD

The study findings revealed that frailty, age, nutritional status, ATS, ATE-Target Max, PTE-Target Max, ADL, IADL, and depression were predictors of OD in community-dwelling older adults. The weakened swallowing muscles in frail older adults lead to poor bolus control, delayed swallowing reflexes, and increased risk of aspiration, making frailty a strong predictor of dysphagia [6, 42–43]. Incorporating balance, endurance, and flexibility exercises can help enhance motor coordination and prevent frailty progression. A decline in muscle mass such sarcopenia and neuromuscular function in older adults lead to impaired swallowing mechanism [7]. Comprehensive geriatric assessment for older adults evaluating physical, nutritional, and functional function could help to identify dysphagia early and timely interventions that maintain better swallowing function, reduce the risk of complications, and improve their overall quality of life. Malnourished older adults may have weakened swallowing muscles, reducing their ability to generate adequate swallowing force [8]. Nutritional interventions including protein and caloric supplementation and dietary modification to support recovery and maintenance of muscle strength could help maintain and improve muscle mass and reduce the risk of malnutrition.

Anterior tongue strength and endurance, emerged as protective factors for dysphagia due to their ability to maintain sustained pressure during swallowing, which is crucial for efficient bolus propulsion and airway protection [9]. Performing tongue strength and endurance training through repetitive exercises could help improve bolus control and reduce the risk of aspiration during swallowing. Poor physical function often correlates with generalized weakness, reduced mobility, and dependence, which are linked to higher risk of dysphagia due to diminished muscular control and coordination [10]. Regular physical activity, including resistance training and aerobic exercises and engaging older adults in self-care can improve their functional independence improving muscle control and coordination. Depression often leads to decreased appetite, leading to poor oral intake, weight loss, and muscle weakness, contributing to swallowing difficulties [11]. Psychosocial interventions including psychological counseling and group therapy can help improve psychological well-being and engagement in health-promoting behaviors, such as proper nutrition leading to better nutritional status. Therefore, routine screening for OD in older adults, along with consistent monitoring of physical function, nutritional status, and mental health, could help in early detection and timely interventions, reducing the OD-associated complications.

### Strengths and limitations

The study has several strengths. First, this is the first study to compare accuracy of IOPI using tongue strength, endurance, and pressure in community-dwelling older adults, who are often underrepresented in dysphagia research. Second, the study examined multiple swallowing parameters, including tongue strength, endurance, and pressure providing a detailed assessment and offering valuable guidance on their appropriate use in community. Third, the study revealed that frailty and tongue endurance as key predictors of OD in older adults suggesting targeted interventions for preventing OD in community-dwelling older adults.

This study has some limitations. Enrollment during the COVID-19 pandemic affected willingness of older adults to participate in the study, contributing to the smaller sample size. Moreover, the small sample size posed challenges for establishing reliable cut-off values, particularly given the small AUC for certain parameters including PTS. As such, caution should be exercised when interpreting these cut-off values. Additionally, the participants were from a specific region, limiting the generalizability of the results, highlighting the diversity of the community sample to ensure representativeness among community-dwelling older adults.

## Conclusions

The study findings demonstrate anterior and posterior tongue strength and endurance to be reliable and effective parameters for early detection of OD using IOPI in community-dwelling older adults. The screening of OD in the community often lacks precise and objective diagnostic tools and the IOPI presents as simple screening tool to improve the accessibility of objective measurements and an effective way of identifying older people at risk of OD in the community. Additionally, the results suggest that frailty, age, nutritional status, ATS, ATE-Target Max, PTE-Target Max, physical function, and depression assessment could facilitate and enhance the identification of community-dwelling older adults at higher risk for OD. Moreover, targeted interventions to improve tongue strength and endurance could help in reducing OD burden in community-dwelling older adults. Overall, IOPI offers a feasible and objective approach for early detection of OD in community-dwelling older adults allowing for timely referral and appropriate care preventing OD-related complications.

## Electronic supplementary material

Below is the link to the electronic supplementary material.

Supplementary Material 1

Supplementary Material 2

## Data Availability

The author confirms that all data generated or analysed during this study are included in this published article and its Supplementary Information.

## Abbreviations

IOPI: Iowa Oral Performance Instrument
OD: Oropharyngeal Dysphagia
ATS: Anterior Tongue Strength
PTS: Posterior Tongue Strength
ATE-Target Sec: Anterior Endurance Target Second
PTE-Target Sec: Posterior Endurance Target Second
ATE-Target Max: Anterior Tongue Endurance Target Maximum
PTE-Target Max: Posterior Tongue Endurance Target Maximum
SSP: Saliva Swallowing Pressure
ESP: Effortful Swallowing Pressure
ADL: Activities of Daily Living
IADL: Instrumental Activities of Daily Living
VFSS: Videofluoroscopic Swallowing Study
FEES: Fiber-Optic Endoscopic Evaluation of Swallowing
EAT-10: Eating Assessment Tool-10
OHAT: Oral Health Assessment Tool
Frailty: Cardiovascular Health Study (for frailty evaluation)
MNA: Mini-Nutritional Assessment
MMSE: Mini-Mental State Examination
GDS: Geriatric Depression Scale
ICC: Intraclass Correlation Coefficient
ROC: Receiver Operating Characteristic
