# Supplementary material for: Accuracy of tongue strength, endurance, and pressure using Iowa oral performance instrument and predictors of dysphagia in community-dwelling older adults: a cross-sectional study
Source: BMC Geriatr. 2025 Mar 24;25:194. doi: 10.1186/s12877-025-05859-z (PMC11931766; doi:10.1186/s12877-025-05859-z)
Supplement: Supplementary file 1 — Supplementary Material 1 [file 12877_2025_5859_MOESM1_ESM.docx]

***Accuracy of tongue strength, endurance, and pressure using Iowa Oral Performance Instrument and predictors of dysphagia in community-dwelling older adults***

Figure S1 ROC curve of Anterior Tongue Strength (ATS) (N = 85)


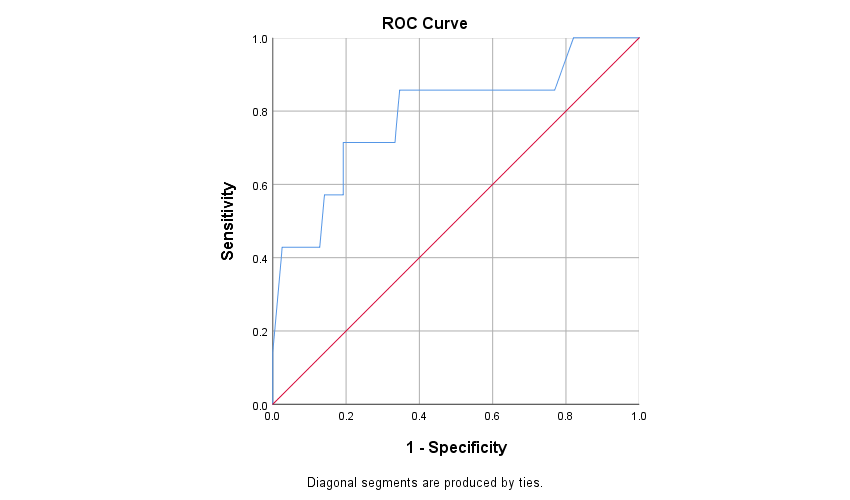


Figure S2 ROC curve of Posterior Tongue Strength (PTS) (N = 85)


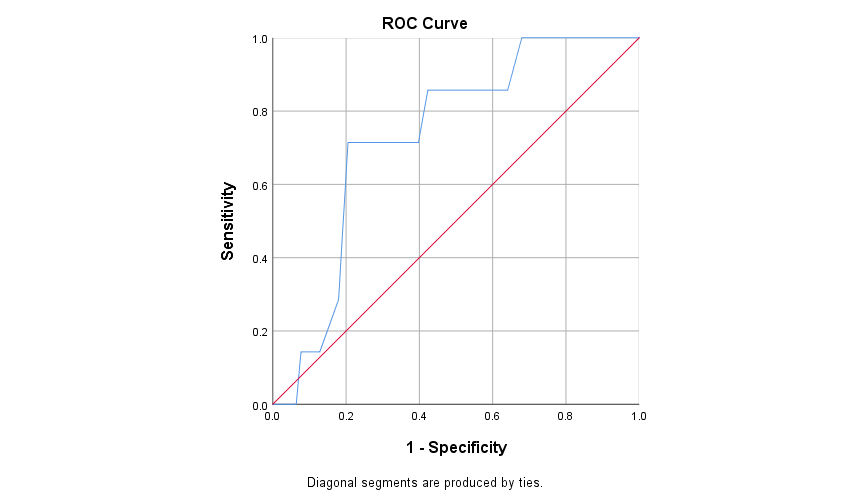


Figure S3 ROC curve of Anterior Tongue Endurance Target Second (ATE -Target Sec) (N = 85)


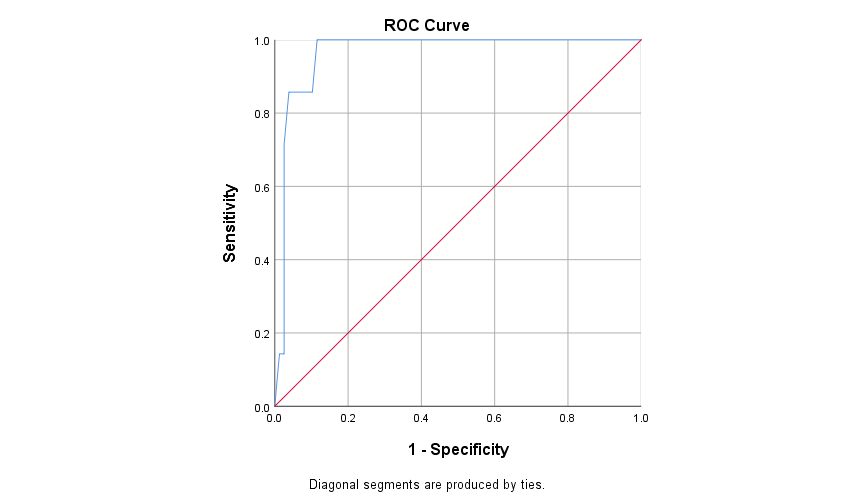


Figure S4 ROC curve of Posterior Tongue Endurance Target Second (PTE- Target Sec) (N = 85)


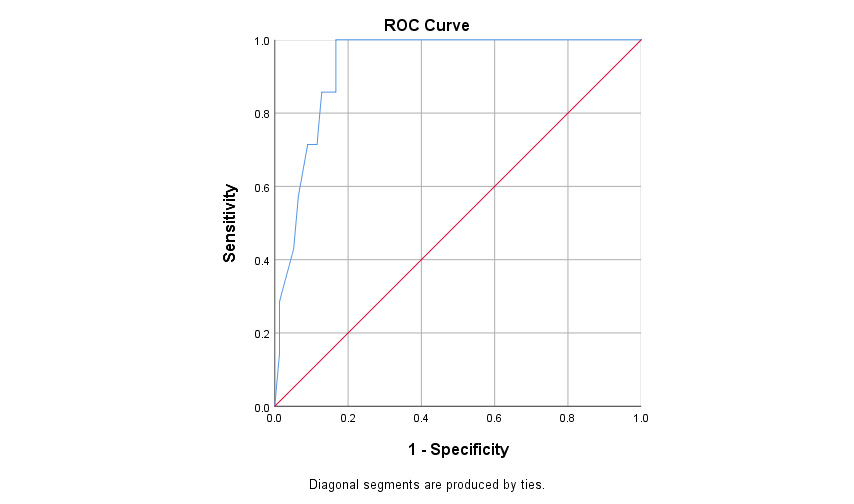


Figure S5 ROC curve of Anterior Tongue Endurance Target Maximum (ATE-Target Max) (N = 85)

| 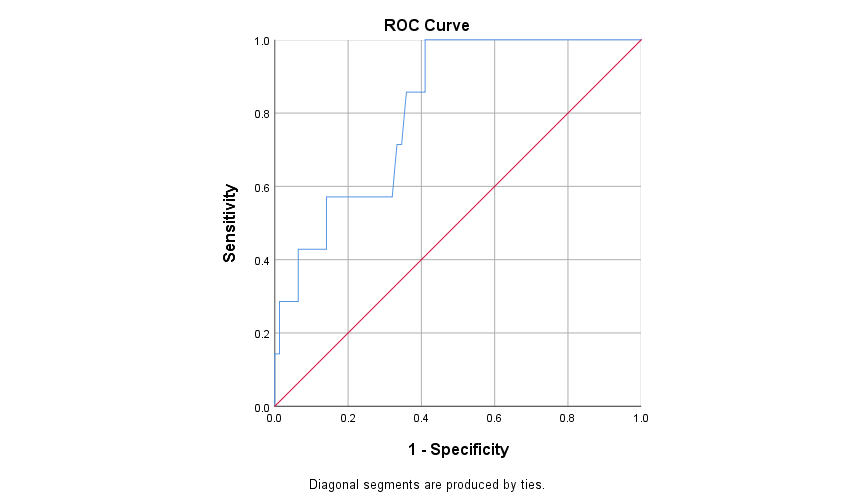 |
| --- |

Figure S5 ROC curve of posterior Tongue Endurance Target Maximum (PTE-Target Max) (N = 85)

| 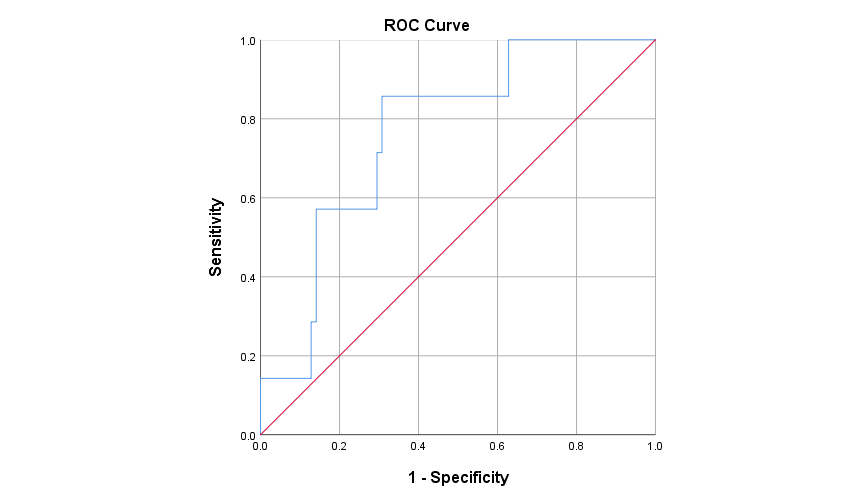 |
| --- |

Figure S7 ROC curve of Saliva swallowing Pressure (SSP) (N = 85)


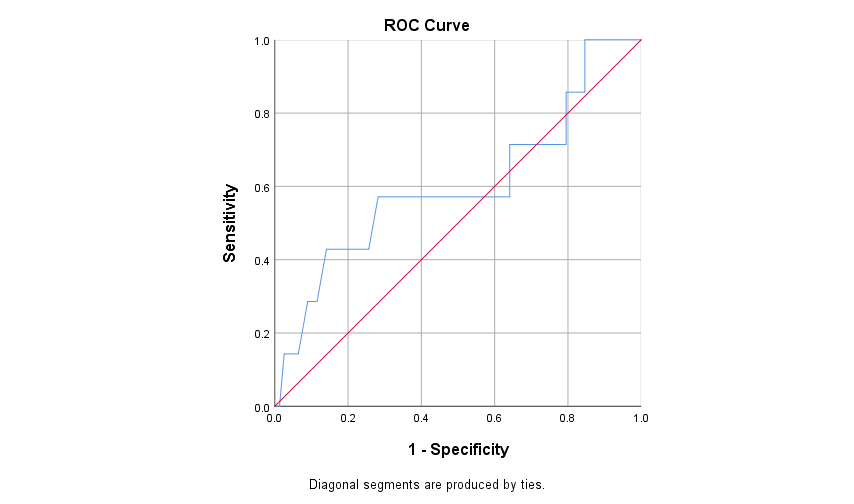


Figure S8 ROC curve of Effortful Swallowing Pressure (ESP) (N = 85)


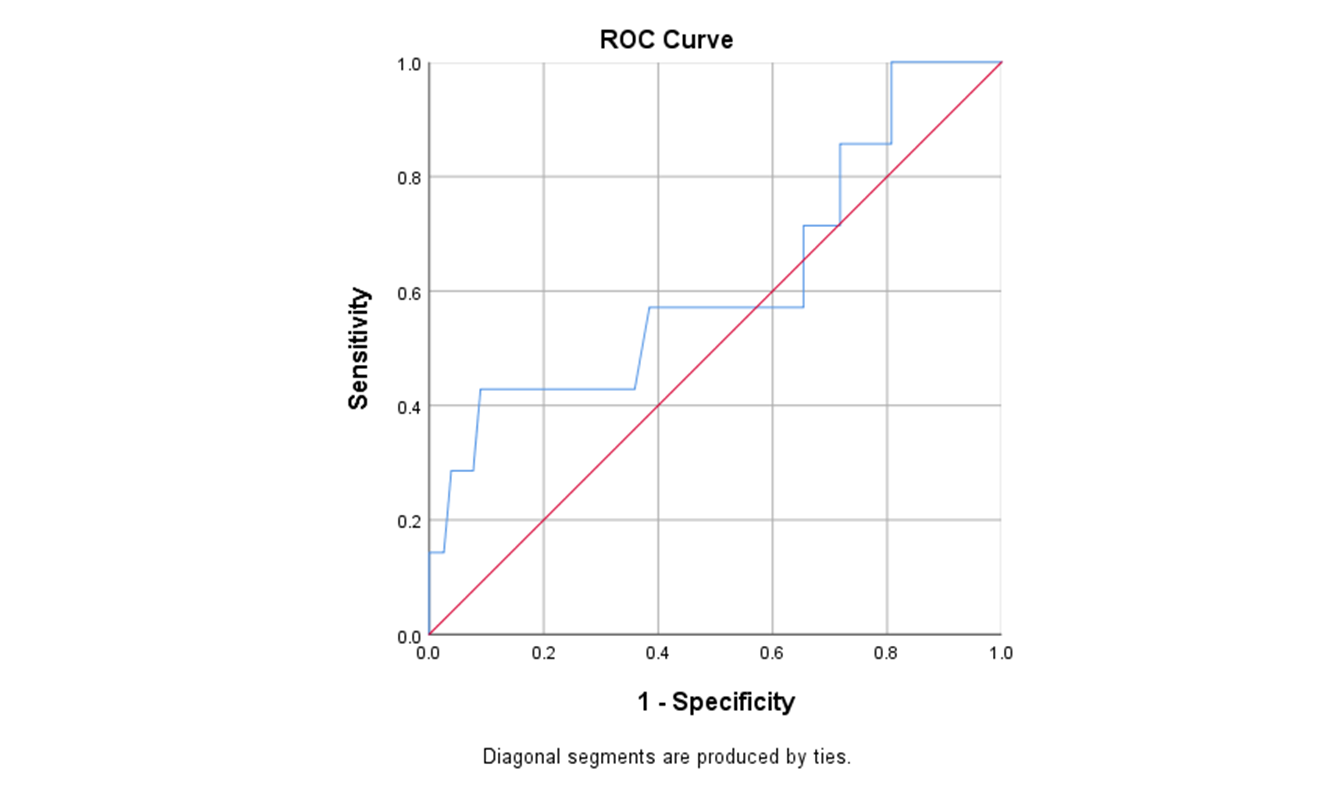


Table S1 Baseline comorbidities of participants with OD (N = 85)

| **Characteristics** | **Total**  **(N = 85)** | **Swallowing Function** | | **P value** |
| --- | --- | --- | --- | --- |
|  |  | **Normal** | **Abnormal** |  |
| Comorbidities, No. (%) | 85 (100) | 78 (100) | 7 (100.0) |  |
| Hypertension^c^ | 56 (65.9) | 52 (66.7) | 4 (57.1) | 0.686 |
| Hyperlipidemia^c^ | 28 (32.9) | 26 (33.3) | 2 (28.6) | 1.000 |
| Sleep disorder^c^ | 26 (30.6) | 22 (28.2) | 4 (57.1) | 0.193 |
| Cataract^c^ | 27 (31.8) | 25 (32.1) | 2 (28.6) | 1.000 |
| Heart disease^c^ | 18 (21.2) | 15 (19.2) | 3 (42.9) | 0.161 |
| Diabetes mellitus^c^ | 19 (22.4) | 17 (21.8) | 2 (28.6) | 0.650 |
| Osteoporosis^c^ | 18 (21.2) | 18 (23.1) | 0 (0.0) | 0.337 |
| BPH^c^ | 12 (14.1) | 9 (11.5) | 3 (42.9) | 0.055 |
| MCI^c^ | 8 (9.4) | 6 (7.7) | 2 (28.6) | 0.129 |
| Arthritis ^c^ | 9 (10.9) | 8 (10.3) | 1 (14.3) | 0.557 |
| Depression^c^ | 7 (8.2) | 6 (7.7) | 1 (14.3) | 0.465 |
| kidney disease^c^ | 7 (8.2) | 7 (9.0) | 0 | 1.000 |
| Glaucoma^c^ | 6 (7.1) | 5 (6.4) | 1 (14.3) | 0.413 |
| Cancer^c^ | 5 (5.9) | 4 (5.1) | 1 (14.3) | 0.356 |

Note: No. (%); ^＊^P < 0.05; ^c^ Fisher’s exact test (chi-squared test).

Abbreviations: OD, Oropharyngeal Dysphagia; BPH, Benign Prostate Hyperplasia; MCI, Mild Cognitive Impairment.

Table S2 Anterior Tongue Strength (ATS): Sensitivity, specificity, and Youden index of different cutoff points (N = 85)

| **Positive if Less Than or Equal To^a^** | **Sensitivity** | **Specificity** | **Youden Index** |
| --- | --- | --- | --- |
| 17.0 | 0.00 | 1.00 | 0.00 |
| 22.0 | 0.14 | 1.00 | 0.14 |
| 26.5 | 0.43 | 0.97 | 0.40 |
| 27.5 | 0.43 | 0.95 | 0.38 |
| 28.5 | 0.43 | 0.90 | 0.33 |
| 29.5 | 0.43 | 0.88 | 0.31 |
| 30.5 | 0.43 | 0.87 | 0.30 |
| 31.5 | 0.57 | 0.86 | 0.43 |
| 32.3 | 0.57 | 0.81 | 0.38 |
| 32.8 | 0.71 | 0.81 | 0.52 |
| 33.5 | 0.71 | 0.78 | 0.50 |
| 34.5 | 0.71 | 0.72 | 0.43 |
| 35.5 | 0.71 | 0.68 | 0.39 |
| 36.5 | 0.71 | 0.67 | 0.38 |
| **37.5*** | **0.86** | **0.65** | **0.51** |
| 38.5 | 0.86 | 0.64 | 0.50 |
| 39.5 | 0.86 | 0.62 | 0.47 |
| 40.5 | 0.86 | 0.56 | 0.42 |
| 41.5 | 0.86 | 0.53 | 0.38 |
| 42.4 | 0.86 | 0.51 | 0.37 |

* Optimal cutoff point

Table S3 Posterior Tongue Strength (PTS): Sensitivity, Specificity, and Youden index of different cutoff points (N = 85)

| **Positive if Less Than or Equal To^a^** | **Sensitivity** | **Specificity** | **Youden Index** |
| --- | --- | --- | --- |
| 17.0 | 0.00 | 1.00 | 0.00 |
| 19.0 | 0.00 | 0.99 | -0.01 |
| 20.5 | 0.00 | 0.97 | -0.03 |
| 22.0 | 0.00 | 0.96 | -0.04 |
| 23.5 | 0.00 | 0.95 | -0.05 |
| 24.5 | 0.00 | 0.94 | -0.06 |
| 25.5 | 0.14 | 0.92 | 0.07 |
| 27.0 | 0.14 | 0.91 | 0.05 |
| 28.5 | 0.14 | 0.90 | 0.04 |
| 29.5 | 0.14 | 0.87 | 0.01 |
| 30.5 | 0.29 | 0.82 | 0.11 |
| **31.5*** | **0.71** | **0.79** | **0.51** |
| 32.5 | 0.71 | 0.78 | 0.50 |
| 33.5 | 0.71 | 0.76 | 0.47 |
| 34.5 | 0.71 | 0.74 | 0.46 |
| 35.5 | 0.71 | 0.73 | 0.45 |
| 36.5 | 0.71 | 0.68 | 0.39 |
| 38.5 | 0.71 | 0.60 | 0.32 |
| 40.0 | 0.86 | 0.58 | 0.43 |
| 41.5 | 0.86 | 0.50 | 0.36 |

* Optimal cutoff point

Table S4 Anterior Tongue Endurance Target Second (ATE-Target Sec): Sensitivity, specificity, and Youden index of different cutoff points (N = 85)

| **Positive if Less Than or Equal To^a^** | **Sensitivity** | **Specificity** | **Youden Index** |
| --- | --- | --- | --- |
| 0.1 | 0.14 | 0.99 | 0.13 |
| 0.3 | 0.14 | 0.97 | 0.12 |
| 0.4 | 0.43 | 0.97 | 0.40 |
| 0.5 | 0.57 | 0.97 | 0.55 |
| 0.8 | 0.71 | 0.97 | 0.69 |
| 1.2 | 0.86 | 0.96 | 0.82 |
| 1.4 | 0.86 | 0.95 | 0.81 |
| 1.8 | 0.86 | 0.94 | 0.79 |
| 2.2 | 0.86 | 0.92 | 0.78 |
| 2.3 | 0.86 | 0.91 | 0.77 |
| **2.4*** | **0.86** | **0.90** | **0.76** |
| 2.8 | 1.00 | 0.88 | 0.88 |
| 3.1 | 1.00 | 0.87 | 0.87 |
| 3.3 | 1.00 | 0.86 | 0.86 |
| 3.5 | 1.00 | 0.85 | 0.85 |
| 3.7 | 1.00 | 0.83 | 0.83 |
| 3.8 | 1.00 | 0.81 | 0.81 |
| 3.9 | 1.00 | 0.79 | 0.79 |
| 4.0 | 1.00 | 0.78 | 0.78 |

* Optimal cutoff point

Table S5 Posterior Tongue Endurance Target Second (PTE-Target Sec): Sensitivity, specificity, and Youden index of different cutoff points (N = 85)

| **Positive if Less Than or Equal To^a^** | **Sensitivity** | **Specificity** | **Youden Index** |
| --- | --- | --- | --- |
| 0.1 | 0.14 | 0.99 | 0.13 |
| 0.2 | 0.29 | 0.99 | 0.27 |
| 0.3 | 0.43 | 0.95 | 0.38 |
| 0.5 | 0.57 | 0.94 | 0.51 |
| 0.6 | 0.71 | 0.91 | 0.62 |
| 0.8 | 0.71 | 0.90 | 0.61 |
| 1.0 | 0.71 | 0.88 | 0.60 |
| 1.1 | 0.86 | 0.87 | 0.73 |
| 1.3 | 0.86 | 0.86 | 0.72 |
| 1.5 | 0.86 | 0.85 | 0.70 |
| **1.7*** | **0.86** | **0.83** | **0.69** |
| 1.9 | 1.00 | 0.83 | 0.83 |
| 2.0 | 1.00 | 0.81 | 0.81 |
| 2.2 | 1.00 | 0.78 | 0.78 |
| 2.3 | 1.00 | 0.74 | 0.74 |
| 2.4 | 1.00 | 0.73 | 0.73 |
| 2.5 | 1.00 | 0.72 | 0.72 |
| 2.6 | 1.00 | 0.69 | 0.69 |
| 2.7 | 1.00 | 0.68 | 0.68 |

* Optimal cutoff point

Table S6 Anterior Tongue Endurance Target Maximum (ATE-Target Max): Sensitivity, specificity, and Youden index of different cutoff points (N = 85)

| **Positive if less than or equal to^a^** | **Sensitivity** | **Specificity** | **Youden index** |  |
| --- | --- | --- | --- | --- |
| 25.9 | 0.57 | 0.83 | 0.40 | |
| 27.2 | 0.57 | 0.81 | 0.38 | |
| 27.8 | 0.57 | 0.79 | 0.37 | |
| 28.0 | 0.57 | 0.78 | 0.35 | |
| 29.1 | 0.57 | 0.77 | 0.34 | |
| 30.1 | 0.57 | 0.76 | 0.33 | |
| 30.7 | 0.57 | 0.74 | 0.32 | |
| 31.3 | 0.57 | 0.73 | 0.30 | |
| 31.8 | 0.57 | 0.72 | 0.29 | |
| 32.1 | 0.57 | 0.71 | 0.28 | |
| 32.4 | 0.57 | 0.69 | 0.26 | |
| 32.9 | 0.57 | 0.68 | 0.25 | |
| 33.5 | 0.71 | 0.67 | 0.38 | |
| 34.1 | 0.71 | 0.65 | 0.37 | |
| **34.4*** | **0.86** | **0.64** | **0.50** | |
| 34.7 | 0.86 | 0.62 | 0.47 | |
| 35.1 | 0.86 | 0.60 | 0.46 | |
| 36.0 | 0.86 | 0.59 | 0.45 | |

* Optimal cutoff point

Table S7 Posterior Tongue Endurance Target Maximum (PTE-Target Max): Sensitivity, specificity, and Youden index of different cutoff points (N = 85)

| **Positive if less than or equal to^a^** | **Sensitivity** | **Specificity** | **Youden Index** |
| --- | --- | --- | --- |
| 25.2 | 0.57 | 0.77 | 0.34 |
| 25.7 | 0.57 | 0.76 | 0.33 |
| 26.3 | 0.57 | 0.74 | 0.32 |
| 27.2 | 0.57 | 0.73 | 0.30 |
| 28.1 | 0.57 | 0.72 | 0.29 |
| 28.4 | 0.57 | 0.71 | 0.28 |
| 28.5 | 0.71 | 0.71 | 0.42 |
| 28.9 | 0.71 | 0.69 | 0.41 |
| **29.5*** | **0.86** | **0.69** | **0.55** |
| 30.1 | 0.86 | 0.68 | 0.54 |
| 30.8 | 0.86 | 0.67 | 0.52 |
| 31.1 | 0.86 | 0.65 | 0.51 |
| 31.3 | 0.86 | 0.63 | 0.49 |
| 31.5 | 0.86 | 0.62 | 0.47 |
| 31.8 | 0.86 | 0.59 | 0.45 |
| 32.1 | 0.86 | 0.58 | 0.43 |
| 32.5 | 0.86 | 0.56 | 0.42 |
| 33.0 | 0.86 | 0.55 | 0.41 |
| 33.3 | 0.86 | 0.54 | 0.40 |
| 33.6 | 0.86 | 0.53 | 0.38 |
| 34.1 | 0.86 | 0.51 | 0.37 |

* Optimal cutoff point

Table S8 Saliva Swallowing Pressure (SSP): Sensitivity, specificity, and Youden index of different cutoff points (N = 85)

| **Positive if Less Than or Equal To^a^** | **Sensitivity** | **Specificity** | **Youden Index** |
| --- | --- | --- | --- |
| 16.4 | 0.29 | 0.90 | 0.18 |
| 16.8 | 0.29 | 0.88 | 0.17 |
| 17.5 | 0.43 | 0.86 | 0.29 |
| 18.5 | 0.43 | 0.85 | 0.27 |
| 20.0 | 0.43 | 0.82 | 0.25 |
| 21.5 | 0.43 | 0.78 | 0.21 |
| 22.5 | 0.43 | 0.74 | 0.17 |
| **23.3*** | **0.57** | **0.72** | **0.29** |
| 23.8 | 0.57 | 0.71 | 0.28 |
| 25.0 | 0.57 | 0.65 | 0.23 |
| 26.5 | 0.57 | 0.62 | 0.19 |
| 27.1 | 0.57 | 0.59 | 0.16 |
| 27.2 | 0.57 | 0.58 | 0.15 |
| 27.5 | 0.57 | 0.56 | 0.14 |
| 27.9 | 0.57 | 0.55 | 0.12 |
| 28.5 | 0.57 | 0.54 | 0.11 |
| 29.5 | 0.57 | 0.51 | 0.08 |
| 30.5 | 0.57 | 0.49 | 0.06 |

* Optimal cutoff point

Table S9 Effortful Swallowing Pressure (ESP): Sensitivity, specificity, and Youden index of different cutoff points (N = 85)

| **Positive if Less Than or Equal To^a^** | **Sensitivity** | **Specificity** | **Youden Index** |
| --- | --- | --- | --- |
| 20.5 | 0.43 | 0.83 | 0.26 |
| 21.5 | 0.43 | 0.81 | 0.24 |
| 23.0 | 0.43 | 0.77 | 0.20 |
| 24.5 | 0.43 | 0.72 | 0.15 |
| 25.2 | 0.43 | 0.69 | 0.12 |
| 25.7 | 0.43 | 0.68 | 0.11 |
| 26.5 | 0.43 | 0.67 | 0.10 |
| 27.5 | 0.43 | 0.64 | 0.07 |
| **28.5*** | **0.57** | **0.62** | **0.19** |
| 29.6 | 0.57 | 0.59 | 0.16 |
| 30.6 | 0.57 | 0.58 | 0.15 |
| 31.5 | 0.57 | 0.55 | 0.12 |
| 32.2 | 0.57 | 0.53 | 0.10 |
| 32.7 | 0.57 | 0.51 | 0.08 |
| 33.1 | 0.57 | 0.47 | 0.05 |
| 33.2 | 0.57 | 0.46 | 0.03 |
| 33.6 | 0.57 | 0.45 | 0.02 |
| 34.0 | 0.57 | 0.44 | 0.01 |
| 34.1 | 0.57 | 0.40 | -0.03 |
| 35.1 | 0.57 | 0.38 | -0.04 |

* Optimal cutoff point
