# Supplementary material for: Accuracy of tongue strength, endurance, and pressure using Iowa oral performance instrument and predictors of dysphagia in community-dwelling older adults: a cross-sectional study
Source: BMC Geriatr. 2025 Mar 24;25:194. doi: 10.1186/s12877-025-05859-z (PMC11931766; doi:10.1186/s12877-025-05859-z)
Supplement: Supplementary file 2 — Supplementary Material 2 [file 12877_2025_5859_MOESM2_ESM.docx]

CONSORT 2010 Flow Diagram

**Analysis (n= 85)**

**Excluded (n=17)**

- **Not meeting inclusion criteria (n= 15)**
- **Declined to participate (n=2)**
- **Other reasons (n=0)**

**Assessed for eligibility (n=102)**

**Complete follow-up (n= 85)**

**Lost to follow-up (n= 0)**

**Enrollment**

**Follow-Up**

**Analysis**

Figure S9. CONSORT 2010 flow diagram of the cross -sectional study.
